# Pipoxolan suppresses the inflammatory factors of NF‐κB, AP‐1, and STATs, but activates the antioxidative factor Nrf2 in LPS‐stimulated RAW 264.7 murine macrophage cells

**DOI:** 10.1002/tox.23000

**Published:** 2020-07-17

**Authors:** Yu‐Hsien Lin, Yu‐Jung Lin, Ting‐Hsuan Chang, Yun‐Hsuan Chang, Yun‐Ping Lim, Jing‐Gung Chung, Wen‐Tsong Hsieh

**Affiliations:** ^1^ School of Pharmacy China Medical University Taichung Taiwan; ^2^ Chang Bing Show‐Chwan Memorial Hospital Changhua Taiwan; ^3^ Graduate Institute of Biomedicine Science China Medical University Taichung Taiwan; ^4^ Department of Psychology, College of Medical and Health Science Asia University Taichung Taiwan; ^5^ Department of Biological Science and Technology China Medical University Taichung Taiwan; ^6^ Department of Biotechnology Asia University Taichung Taiwan; ^7^ Department of Pharmacology China Medical University Taichung Taiwan; ^8^ Chinese Medicine Research Center China Medical University Taichung Taiwan; ^9^ Drug Development Center China Medical University Taichung Taiwan

**Keywords:** anti‐inflammatory effects, antioxidative activity, pipoxolan (PIPO), RAW 264.7 cells, transcription factor

## Abstract

Although pipoxolan (PIPO) is a smooth muscle relaxant, its anti‐inflammatory capability has not been studied. Therefore, we investigated the anti‐inflammatory molecular mechanisms of PIPO in lipopolysaccharide (LPS)‐induced RAW 264.7 macrophages. In this study, we used the MTT assay to evaluate the cytotoxicity, applied the enzyme‐linked immunosorbent assay to determine the inflammatory cytokines, and performed Western blotting to assess protein expression. The results showed that PIPO significantly inhibited cytokine production, including nitric oxide, prostaglandin E_2_, tumor necrosis factor‐α, and interleukin‐6. PIPO also suppressed the pro‐inflammatory mediator expression with inducible nitric oxide synthase and cyclooxygenase‐2. Moreover, PIPO prohibited the multiple inflammatory transcription factor pathways, including inhibitor kappa B/nuclear factor of the κ light chain enhancer of B cells (NF‐κB), mitogen‐activated protein kinase/activator protein‐1 (AP‐1), Janus kinase/signal transducer and activator of transcription (STAT), and toll‐like receptor 4 (TLR4)/serine/threonine kinase (AKT). Besides, PIPO effectively activated the nuclear factor erythroid 2‐related factor 2 (Nrf2)/heme oxygenase‐1 antioxidative pathway. Collectively, PIPO may attenuate the inflammatory effects via influencing the LPS/TLR4 receptor binding; suppress the expression of anti‐inflammatory transcription factors NF‐κB, AP‐1, and STAT; and activating the antioxidative transcription factor Nrf2 in LPS‐stimulated mouse RAW 264.7 cells.

## 1 INTRODUCTION

Inflammation is a defensive immune response of the animal body to stimuli. However, uncontrolled inflammation can lead to immunodeficiency and many chronic diseases.^1^ In mammals, the most inflammatory process always initiated from lipopolysaccharide (LPS) binding to the TLR4 coreceptor.^2^ During the inflammatory process, LPS binds to the transmembrane toll‐like receptor 4 (TLR4) receptor and activates the myeloid differentiation primary response 88 (MyD88)‐dependent or/and independent pathways. Through activating the Akt/MAPK pathway by (MyD88)‐dependent activation, the transcription factors NF‐κB and AP‐1 translocate from the cytoplasm to the nucleus, leading to the production of inflammatory cytokines such as IL‐1, IL‐6, and TNF‐α.^3^ In contrast, the activated MyD88 independent pathway can prompt the interferon regulatory factor signal pathway, which enables the secondary wave of transcription factor STATs to transfer from the cytoplasm into the nucleus.^4^

NF‐κB, the multipotent transcriptional factor, plays a critical role in the pro‐inflammatory processes.^5^ Phosphorylated inhibition of kappa B (IκB) is the crucial step in starting the nuclear translocation of NF‐κB from cytoplasm to the nucleus, which can regulate the transcription and expression of multiple genes for inflammation and immune responses.^6^ Besides, the MAPK pathway regulates transcription and translation levels of inflammatory mediators by activating transcription factors AP‐1 and NF‐κB.^7^ Abnormal activation of transcription factors AP‐1 (c‐Jun, c‐Fos, Jun D, and ATF2) is associated with redox‐mediated inflammation and related to the transcription of pro‐inflammatory genes encoding enzymes (such as iNOS, COX‐2).^8^, ^9^ In chronic inflammatory responses, the Janus kinase (JAK)/STAT signaling triggered the pro‐inflammatory cytokines (TNF‐α, IL‐2, and IL‐6).^8^

JAKs are receptor tyrosine kinases that mediate phosphorylation of STATs. The activated STAT dimerizes and translocates to the nucleus and binds to the promoter of the target gene, which then regulates its transcription.^9^ It has demonstrated that Nrf2 blocks the expression of pro‐inflammatory cytokines and prevents inflammatory responses, Nrf2 cascade acts as a novel anti‐inflammatory signal to heme oxygenase‐1 (HO‐1) expression in macrophages.^10^ Moreover, MAPKs also assist TLR signaling in cross talking with the Nrf2 pathway.^11^ Thus, Nrf2 can translocate into the nucleus and initiates the transcription of genes encoding cytoprotective proteins. Among these proteins, HO‐1, superoxide dismutase (SOD), and catalase (CAT) play a vital role in anti‐inflammation and antioxidation responses.^12^

**FIGURE 1:**
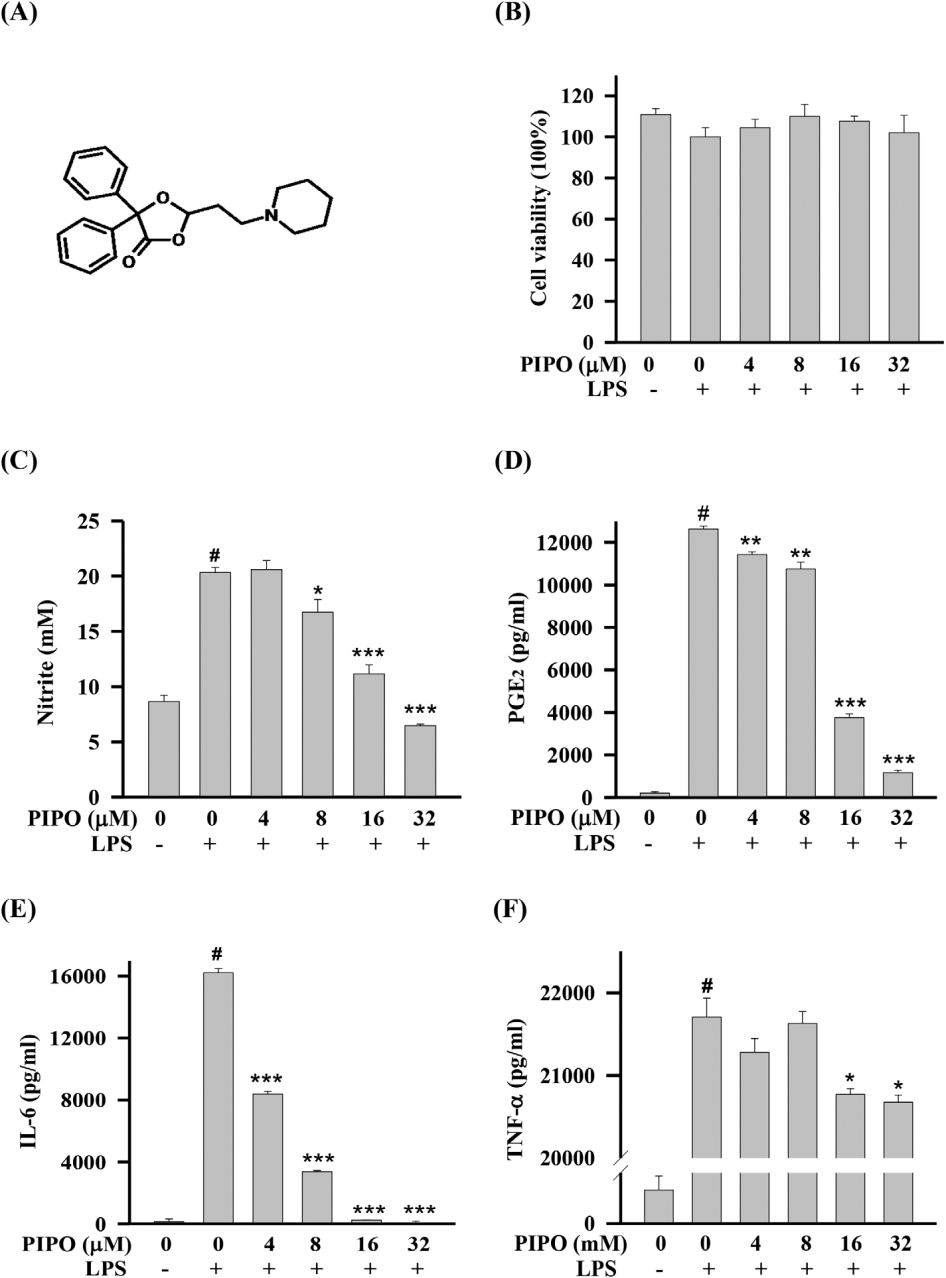
Cell viability and pipoxolan (PIPO) inhibited the ratio of inflammatory cytokines production in lipopolysaccharide (LPS)‐stimulated RAW264.7 cells. (A) The chemical structure of PIPO. RAW264.7 cells were pretreated with PIPO (0, 4, 8, 16, and 32 μM) for 1 hour then stimulated with LPS (100 ng/mL) for 24 hours. We then collected suspension media to quantify the production of cytokines and analyzed the cytotoxicity in the remaining cells. (B) In the remaining cells, we analyzed the cytotoxicity by the MTT assay. (C) In the suspension media, we detected the nitrite production with the Griess reaction assay. We measured the cytokines production of prostaglandin E_2_ (PGE_2_) (D), interleukin‐6 (IL‐6) (E), and tumor necrosis factor‐α (TNF‐α) (F) by using the specific EIA kit, respectively (n = 3). Data are presented as mean ± SE of three independent experiments. #*P* < .05 compared with the negative LPS‐stimulated group, *P < .05, **P < .01, and ***P < .001 compared with the LPS group

Pipoxolan (PIPO) (Figure 1A) is a smooth muscle relaxant that inhibits phosphodiesterase and maintenance cyclic‐AMP levels and prevents Ca^2+^ entering the cell membrane in smooth muscle.^13^, ^14^ According to earlier studies, PIPO can inhibit the phosphorylation of JNK and p38 and inhibit the expression metalloproteinases‐2 (MMP‐2) and MMP‐9.^15^ PIPO also inhibited the phosphatidylinositol‐4,5‐diphosphate 3‐kinase (PI3K) protein expression, and the phosphorylation of protein kinase B (AKT), indicating that PIPO inhibits PI3K/AKT intrinsic apoptotic signaling pathways.^16^ Moreover, PIPO can also inhibit the Ras/MEK/ERK pathway preventing neuronal apoptosis, decreasing the migration and intimal thickening in vascular smooth muscle cells.^13^ Therefore, this study evaluated the anti‐inflammatory molecular mechanism of PIPO in inflammatory transcription factor and antioxidant transcription factor in LPS‐stimulated mouse RAW264.7 cells.

## 2 MATERIALS AND METHODS

### 2.1 Chemicals and reagents

PIPO was a gift from the Wide Pharmaceutical Co., Ltd. (Taichung, Taiwan). LPS (lipopolysaccharides from *Escherichia coli* O111:B4), 3‐(4, 5‐dimethylthiazol‐2‐yl)‐2,5‐diphenyltetrazolium bromide (MTT), Griess reagent (1% sulfanilamide, 0.1% N‐1‐naphthylenediamine dihydrochloride, and 2.5% phosphoric acid), cell lysis buffer, and dimethyl sulfoxide were obtained from Sigma‐Aldrich (St. Louis, Missouri). Dulbecco's modified Eagle medium (DMEM) and fetal bovine serum (FBS) were obtained from Life/Gibco (Grand Island, New York). Antibodies against iNOS, COX‐2, NF‐κB/p65, NF‐κB/p50, Nrf2, HO‐1, p‐STAT1 (705), STAT1, p‐STAT3 (727), and p‐STAT3 (705), and STAT3 were obtained from Cell Signaling (Beverly, Massachusetts). TRIzol reagent, SuperScript II Reverse Transcriptase, RNaseOUT Recombinant RNase Inhibitor, Hoechst 33258, and Alexa Fluor 488 or 568 were obtained from Invitrogen (Carlsbad, California). Prostaglandin E2 ELISA Kit was from Cayman (Ann Arbor, Michigan).

### 2.2 Cell line and culture

RAW 264.7 murine macrophage cell lines were obtained from the Food Industry Research and Development Institute (Hsinchu, Taiwan). Cells were cultured in DMEM medium supplemented with 10% FBS and antibiotics (100 units/mL penicillin and 100 μg/mL streptomycin) at 37°C in a humidified incubator of 5% CO_2_.^14^

### 2.3 MTT assay

Cell viability of RAW 264.7 macrophages was determined using the MTT assay.^17^ RAW264.7 cells (3 × 10^4^/well) were seeded in 96‐well cell culture plates overnight. Cells were treated with various concentrations of PIPO (0, 4, 8, 16, and 32 μM) for 1 hour and then incubated with LPS (100 ng/mL) for 24 hours. Subsequently, the culture medium was removed and incubated with 100 μL of 0.5 μM MTT reagent for 2 hours at 37°C. The culture medium was removed and 100 μL of 0.04 N HCl/isopropyl alcohol was added. The absorbance at 570 nm was measured with a Micro‐Reader EPOCH2 plate reader (BioTek, USA).

### 2.4 Nitrite assay

The production of nitrite was determined using Griess reagent.^18^ RAW264.7 cells (3 × 10^4^/well) were seeded in 96‐well cell culture plates overnight. Cells were treated with various concentrations of PIPO for 1 hour before incubating with LPS for 24 hours. Cell culture supernatants were collected and, subsequently, nitrite production was detected using the Griess reagent. The absorbance at 540 nm was measured with a Micro‐Reader EPOCH2 plate reader.

### 2.5 Enzyme‐linked immunosorbent assay

The cytokines were produced according to the manufacturer's instructions.^19^ RAW264.7 cells (3 × 10^4^/well) were seeded in 96‐well cell culture plates overnight. Cells were treated with various concentrations of PIPO for 1 hour before incubating with LPS. Cell culture supernatants were collected and subsequently, the levels of cytokines (TNF‐α, IL‐1β, and IL‐6) were detected using the enzyme‐linked immunosorbent assay (ELISA) kits. The absorbance was measured with a Micro‐Reader EPOCH2 plate reader.

### 2.6 Real‐time PCR

The total RNA was extracted using a TRIzol reagent kit according to the manufacturer's instructions.^20^ RAW 264.7 cells (1.5 × 10^5^ cells/well) in six‐well plates were pretreated with various concentrations of PIPO for 1 hour before incubating with LPS. Total RNA extracted by TRIzol reagent was reverse‐transcribed into DNA by using the cDNA Reverse Transcription Kit. SYBR Green Master Mix was used to perform PCR reaction by StepOne Plus Real‐Time PCR Systems under the following conditions: 95°C for 10 minutes, 42 cycles at 95°C for 10 seconds, and then 60°C for 60 seconds. The following primers were used for iNOS, sense primer, 5′‐AGCAACTACTGCTGGTGGT‐3′, antisense primer, 5′‐AATGGGCAGACTCTGAAGA‐3′; for COX‐2, sense primer, 5′‐CTGGAACATGGACTCACTCAGTTTGT‐3′, antisense primer, 5′‐ACAAGCAGTGGCAAAGGCCT‐3′; and for GAPDH, sense primer, 5′‐GGCCTT‐CCGTGTTCCTACC‐3′, antisense primer, 5′‐GAAGGTGGTGAAGCAGGCA −3′. The results were expressed as the ratio of optical density to GAPDH.

### 2.7 Western blotting assay

Total protein or nuclear protein extracts from cells were prepared as described previously.^21^ RAW 264.7 cells were pretreated with PIPO for 1 hour before incubating with LPS. The cells were extracted the total proteins with PRO‐PREP Protein Extraction Solution (iNtRON, Seoul, Korea). Proteins were separated by SDS‐PAGE on 8% to 12% sodium dodecyl sulfate‐polyacrylamide gel and transferred to polyvinylidene fluoride membrane. Subsequently, the membranes were blocked with 5% albumin for 1 hour and probed with primary antibodies overnight at 4°C. The membrane was treated with the corresponding secondary antibodies conjugated with horseradish peroxidase for 1 hour at room temperature. The membranes were washed three times, and the immunoreactive proteins were detected by enhanced chemiluminescence by using ECL reagent (Perking Elmer). Western blot analysis was quantified using the GE Las 4000 Mimi Molecular Imaging System (GE) and the TotalLab gel analysis software (BioSystematica, UK).

### 2.8 Immunofluorescence assay

Immunofluorescence assay was applied to detect the expression with confocal spectral microscopy, as described previously.^22^ RAW 264.7 cells were seeded onto glass coverslips overnight and treated with PIPO for 1 hour before incubating with LPS. After exposure, cells were treated with cold 4% paraformaldehyde for 20 minutes and permeabilized using 0.5% Triton‐X 100 for 30 minutes. Then, the cells were blocked with 5% bovine serum albumin for 1 hour and incubated with a primary antibody of p65, p50, c‐Jun, c‐Fos, p‐Nrf2, p‐STAT3 for overnight at 4°C. The cells were washed three times and incubated with a secondary antibody labeled with Alexa Fluor‐594 for 1 hour. The cells were then washed another three times and DAPI (50 μg/mL) was added for 20 minutes at 37°C in the dark. The coverslips were sealed and the images were taken with a Leica TCS SP2 confocal spectral microscopy.

### 2.9 Statistical analysis

The data were presented as mean ± SE and analyzed using one‐way analysis of variance, and differences between the LPS‐treated and LPS‐untreated (control) groups were considered statistically significant at the level of #*P* < 0.05 vs negative LPS‐stimulated group, *P < .05, **P < .01, and ***P < .001 the PIPO‐treated vs LPS‐stimulated and PIPO‐untreated group.

## 3 RESULTS

### 3.1 Cell viability and PIPO inhibited the rate of inflammatory cytokines production in LPS‐stimulated RAW264.7 cells

In the inflammatory process, LPS binds to the TLR4 receptor and triggers the production of inflammatory cytokines such as IL‐1, IL‐6, and TNF‐α.^3^ According to the current dose, PIPO (Figure 1A) shows no significant cytotoxicity in RAW264.7 cells (Figure 1B). However, PIPO suppresses the inflammatory effects by inhibiting the cytokine production of nitrite (Figure 1C), PGE_2_ (Figure 1D), IL‐6 (Figure 1E), and TNF‐α level (Figure 1F) in LPS‐stimulated RAW264.7 cells. In RAW 264.7 cells, the concentration at 32 μM of PIPO inhibited the rate of inflammatory cytokines production on NO, PGE2, TNF‐α, and IL‐6 were 68.24%, 90.73%, 90%, and 4.73%, respectively.

### 3.2 PIPO inhibited the proteins and mRNA expression of the pro‐inflammatory mediator iNOS and COX‐2 in LPS‐stimulated RAW264.7 cells

**FIGURE 2:**
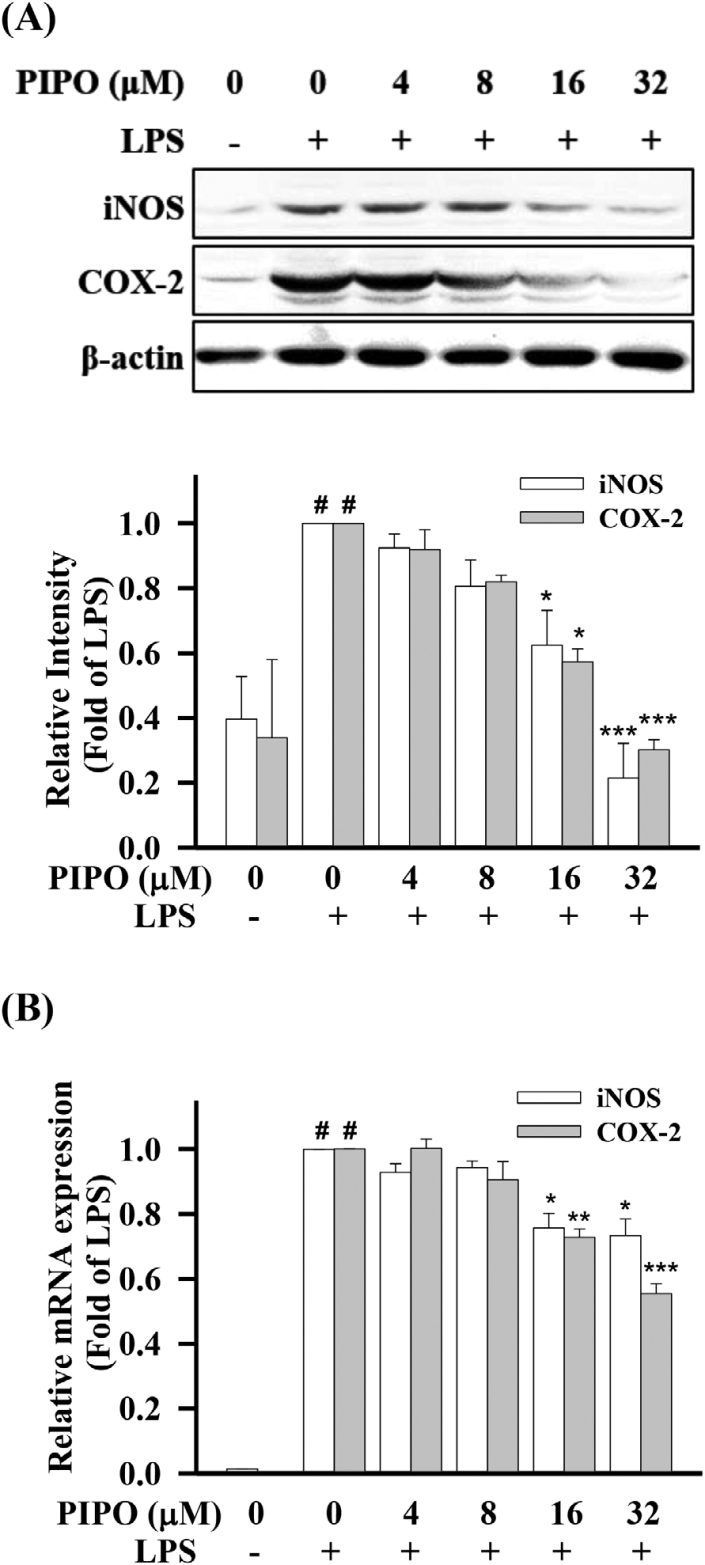
Pipoxolan (PIPO) inhibited the proteins and messenger RNA (mRNA) expression of the pro‐inflammatory mediator inducible nitric oxide synthase (iNOS) and cyclooxygenase‐2 (COX‐2) in lipopolysaccharide (LPS)‐stimulated RAW264.7 cells. RAW264.7 cells were pretreated with PIPO (0, 4, 8, 16, and 32 μM) for 1 hour and then stimulated with LPS (100 ng/mL) for 24 hours. (A) Measuring the protein expressions of pro‐inflammatory mediators iNOS and COX‐2 by western blotting (n = 3). (B) Detecting the relative mRNA expression of pro‐inflammatory mediators iNOS and COX‐2 by real‐time quantitative PCR (qRT‐PCR) (n = 3). Data are presented as mean ± SE. #*P* < .05 compared with the negative LPS‐stimulated group, *P < .05, **P < .01, and ***P < .001 compared with the LPS group

NF‐κB regulates inflammation‐related enzymes such as iNOS and COX‐2 to initiate the production of various inflammatory cytokines (TNF‐α, IL‐6, and IL‐1β), chemokines, and immune gene expression.^23^, ^24^ Abnormal activation of transcription factor triggers pro‐inflammatory genes encoding enzyme expression (such as iNOS and COX‐2).^25^ According to the results, we found that LPS could increase the pro‐inflammatory mediator expression of iNOS and COX‐2 in RAW264.7 cells. However, PIPO suppressed the pro‐inflammatory protein expression of iNOS and COX‐2 in LPS‐stimulated RAW264.7 cells (Figure 2A). Moreover, PIPO also significantly inhibited the mRNA expression of iNOS and COX‐2 in the LPS‐stimulated manner (Figure 2B).

### 3.3 PIPO inhibited the phosphorylation of IKKα/β and IκB and inhibited the nuclear translocation of NF‐κB in LPS‐stimulated RAW264.7 cells

**FIGURE 3:**
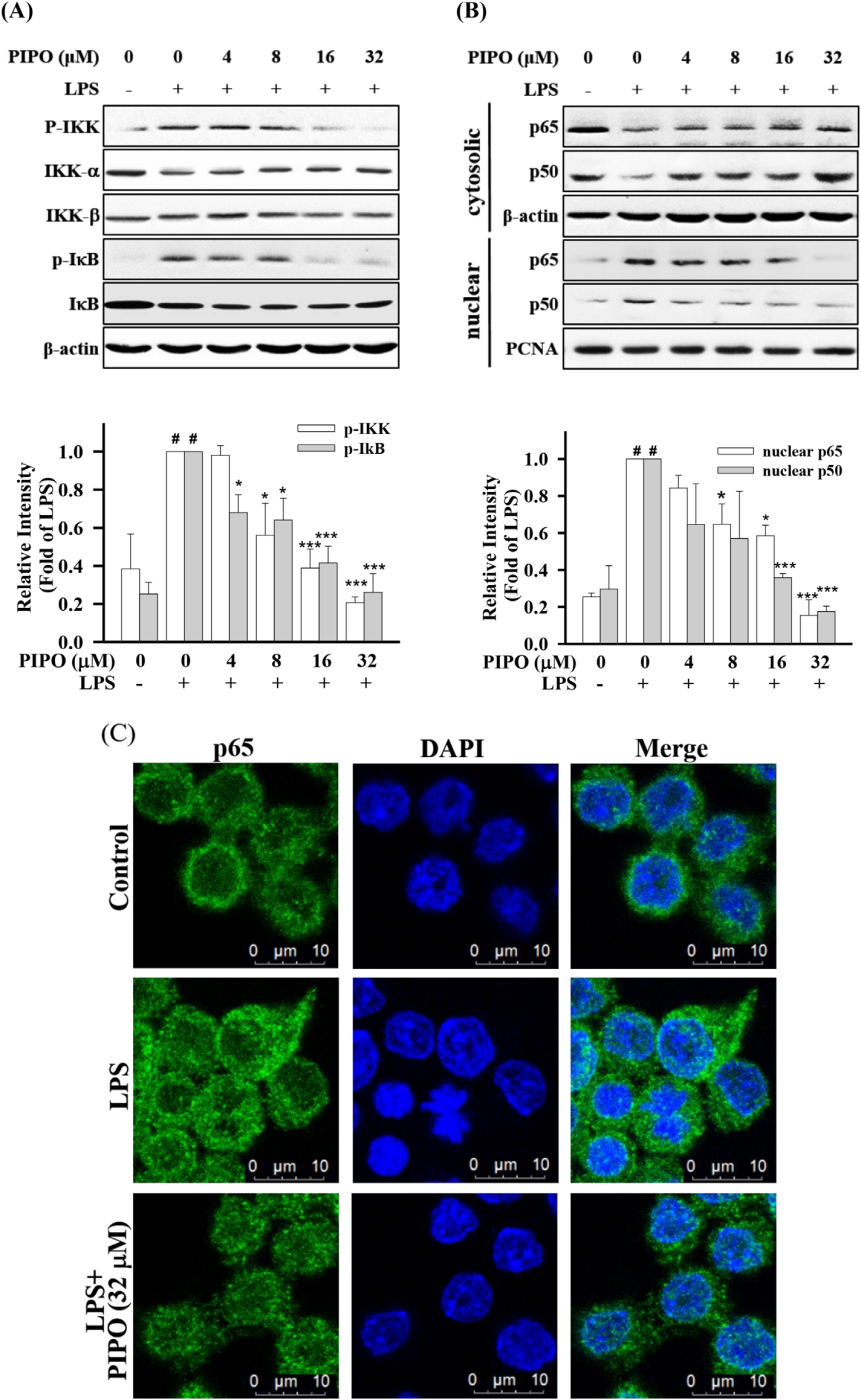
Pipoxolan (PIPO) inhibited the phosphorylation of IKKα/β and inhibitor kappa B (IκB) and inhibited the nuclear translocation of NF‐κB in lipopolysaccharide (LPS)‐stimulated RAW264.7 cells. RAW264.7 were cells pretreated with PIPO (0, 4, 8, 16, and 32 μM) and then treated with LPS (100 ng/mL) for 30 minutes. Then, (A) the protein expression of p‐IKKα/β and p‐IκBα was detected by Western blotting (n = 3), (B) the nuclear translocation of transcription factor NF‐κB p65 and p50 from the cytoplasm to the nucleus was measured by Western blotting (n = 3). (C). RAW264.7 cells were pretreated with PIPO (32 μM) for 1 hour and then stimulated with LPS (100 ng/mL) for 2 hours. The localization of p65 was measured by immunofluorescence staining from the cytoplasm to the nucleus. Data are presented as mean ± SE. #*P* < .05 compared with the negative LPS‐stimulated group, *P < .05, **P < .01, and ***P < .001 compared with the LPS group [Color figure can be viewed at wileyonlinelibrary.com]

In the natural state, NF‐κB always stabilized in the cytoplasm by binding to the IκB junction. IKKβ is an IκB kinase and an essential factor in NF‐βB activity.^26^ The results demonstrated that treatment with PIPO inhibited LPS‐stimulated IKK phosphorylation and IκBα phosphorylation, respectively (Figure 3A). However, PIPO inhibited the LPS‐stimulated nuclear translocation of p65 and p50 subunit in macrophages (Figure 3B). Next, LPS significantly activated cytosolic p65 and p50 and decreased nuclear p65 and p50 (Figure 3B). Moreover, in LPS‐stimulated RAW264.7 cells, immunofluorescence analysis indicated that PIPO suppressed the translocation of NF‐κB/p65 into the nucleus (Figure 3C).

### 3.4 PIPO suppressed the phosphorylation of MAPK and inhibited the nuclear translocation of AP‐1 in LPS‐stimulated macrophages RAW264.7 cells

**FIGURE 4:**
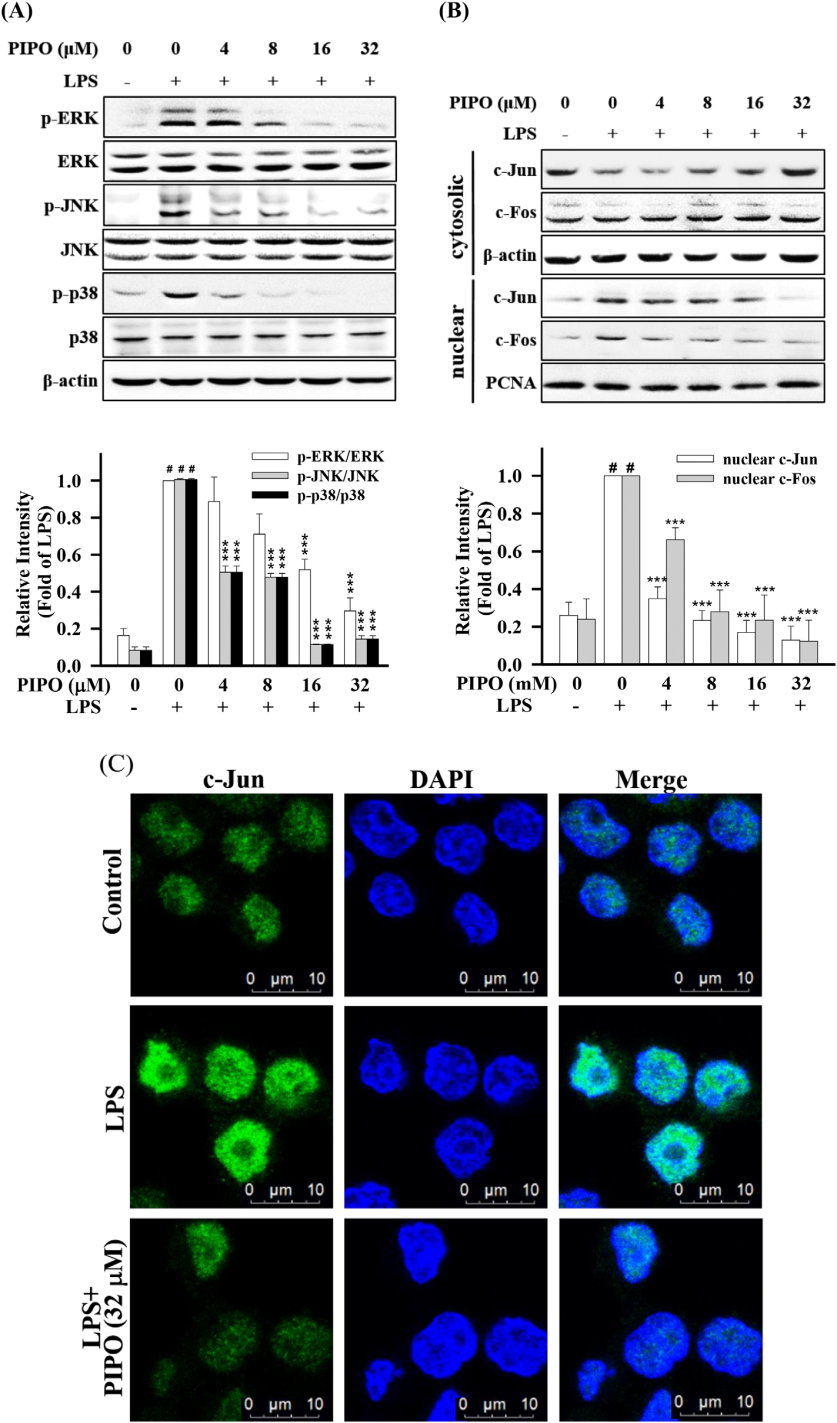
Pipoxolan (PIPO) suppressed the phosphorylation of mitogen‐activated protein kinase (MAPK) and inhibited the nuclear translocation of AP‐1 in lipopolysaccharide (LPS)‐stimulated macrophages RAW264.7 cells. RAW 264.7 cells were pretreated with PIPO (0, 4, 8, 16, and 32 μM) for 1 hour and then stimulated with LPS (100 ng/mL) for 30 minutes. (A) The protein expression of p‐ERK, p‐JNK, and p‐p38 MAPK was analyzed, (B) the protein expression of transcription factor c‐Jun and c‐Fos in cytoplasm and nucleus by western blotting (n = 3). (C). RAW264.7 cells were pretreated with PIPO (32 μM) for 1 hour and then stimulated with LPS (100 ng/mL) for 2 hours. The localization of c‐Jun in the cytoplasm and nucleus measured by immunofluorescence staining. As for control, cells were incubated without LPS and PIPO. Data are presented as mean ± SE. #*P* < 0.05 compared with the negative LPS‐stimulated group, *P < .05, **P < .01, and ***P < .001 compared with the LPS group [Color figure can be viewed at wileyonlinelibrary.com]

AP‐1 is an important inflammation‐related transcription factor. When LPS is induced, MAPK phosphorylation can promote AP‐1 translocate into the nucleus and increase the production of pro‐inflammatory cytokines and chemokines.^27^ Therefore, we examined the expression of phosphorylation of ERK1/2, JNK, p38MAPK, and the translocation of AP‐1 (c‐Jun and c‐Fos) in LPS‐stimulated RAW 264.7 cells. As shown in Figure 4A, LPS improved the expression of p‐JNK, p‐ERK, and p‐p38 in LPS‐stimulated RAW264.7 cells. However, PIPO could suppress the expression of p‐JNK, p‐p38, and p‐ERK. Furthermore, LPS significantly increased cytosolic c‐Jun and c‐Fos, but the activation was significantly suppressed by pretreating with PIPO (Figure 4B). Furthermore, the immunofluorescence staining showed that PIPO suppressed nuclear c‐Jun expression in LPS‐stimulated RAW264.7 cells (Figure 4C). These collective data indicate that PIPO could inhibit the activation of MAPK signaling pathways and suppressed the nuclear translocation of c‐Jun and c‐Fos into the nucleus.

### 3.5 PIPO suppressed the phosphorylation of JAK 1/2 and STAT1/3 in LPS‐stimulated macrophages RAW264.7

**FIGURE 5:**
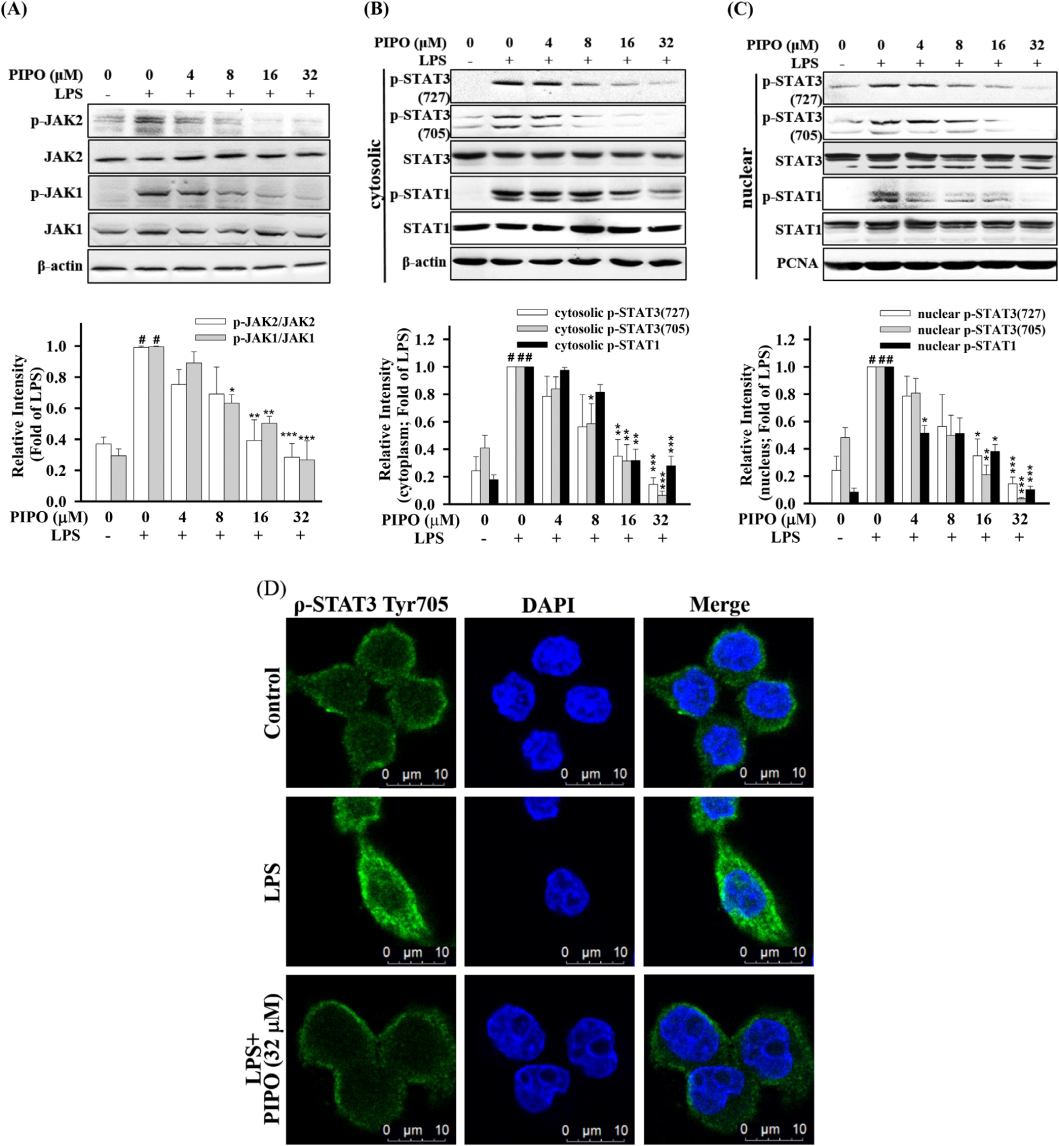
Pipoxolan (PIPO) suppressed the phosphorylation of JAK1/2 and STAT1/3 in lipopolysaccharide (LPS)‐stimulated macrophages RAW264.7. RAW 264.7 cells were pretreated with PIPO (0, 4, 8, 16, and 32 μM) for 1 hour and then stimulated with LPS (100 ng/mL) for 6 hours. Next, (A) the phosphorylation of JAK1/2 was analyzed by western blotting (n = 3), (B) in the cytoplasm, the protein expression of p‐STAT1 (705), p‐STAT3 (727), and p‐STAT3 (705) was analyzed (n = 3), and (C) in the nucleus, the protein expression of p‐STAT1 (705), p‐STAT3 (727), and p‐STAT3 (705) was analyzed (n = 3). (D) RAW264.7 cells were pretreated with PIPO (32 μM) for 2 hours and then stimulated with LPS (100 ng/mL) for 6 hours. The localization of p‐STAT (Tyr705) in the cytoplasm and nucleus were measured by immunofluorescence staining. As for control, cells were incubated without LPS and PIPO. Data are presented as mean ± SE. #*P* < .05 compared with the negative LPS‐stimulated group, *P < .05, **P < .01, and ***P < .001 compared with the LPS group [Color figure can be viewed at wileyonlinelibrary.com]

In macrophages, STAT1 regulates the TNF‐α‐dependent expression of transporter.^28^ As shown in Figure 5A, PIPO inhibited the phosphorylation and expression of JAK1/2 in LPS‐stimulated macrophages RAW264.7 cells. Therefore, in the cytoplasm, PIPO significantly decreased the LPS‐stimulated phosphorylation and protein expression of p‐STAT1 (705), p‐STAT3 (727), and p‐STAT3 (705) (Figure 5B). Moreover, in the nucleus, PIPO also significantly reduced the LPS‐stimulated translocation and protein expression of p‐STAT1, p‐STAT3 (727), and p‐STAT3 (705) (Figure 5C). Furthermore, according to the immune‐fluorescence analysis, it is indicated that PIPO suppressed the translocation of p‐STAT3 (Tyr705) into the nucleus in LPS‐stimulated RAW264.7 cells (Figure 5D).

### 3.6 PIPO suppressed the LPS‐stimulated inflammatory response through regulated Nrf2/HO‐1 pathway

**FIGURE 6:**
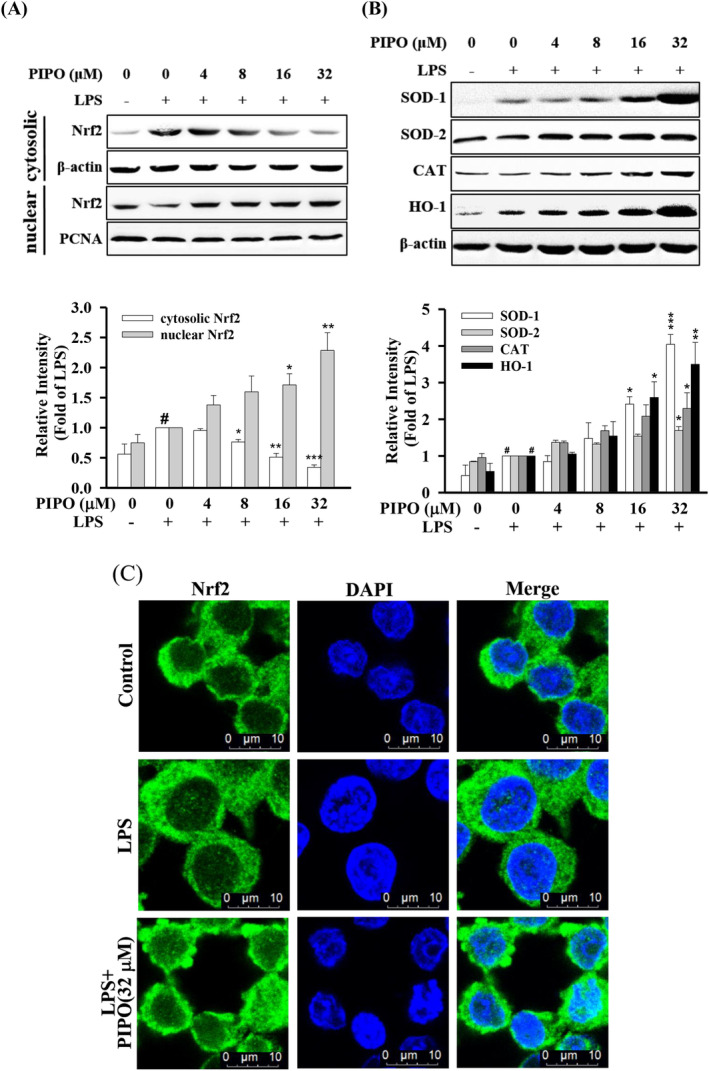
Pipoxolan (PIPO) suppressed the lipopolysaccharide (LPS)‐stimulated inflammatory response by regulating the nuclear factor erythroid 2‐related factor 2 (Nrf2)/heme oxygenase‐1 (HO‐1) pathway. RAW264.7 cells were pretreated with PIPO (0, 4, 8, 16, and 32 μM) for 1 hour and then stimulated with LPS (100 ng/mL) for 6 hours. (A). The protein expression of the transcription factor Nrf2 in the cytoplasm and nucleus measured by western blotting. (B) RAW264.7 cells were pretreated with PIPO (0, 4, 8, 16, and 32 μM) for 1 hour and then stimulated with LPS (100 ng/mL) for 24 hours, then protein expression of HO‐1, SOD1, SOD2, and catalase (CAT) was measured by Western blotting (n = 3). (C) RAW264.7 cells pretreated with PIPO (32 μM) for 1 hour and then stimulated with LPS (100 ng/mL) for 6 hours, then the protein expression of Nrf2 was measured by immunofluorescence staining. Data are presented as mean ± SE. #*P* < .05 compared with the negative LPS‐stimulated group, *P < .05, **P < .01, and ***P < .001 compared with the LPS group [Color figure can be viewed at wileyonlinelibrary.com]

Nrf2/HO‐1 is a critical antioxidant pathway, and the transcription factor Nrf2 suppresses oxidative stress and regulates anti‐inflammatory.^29^ Moreover, Nrf2 is correlated with the induction of HO‐1, GPx, and glutathione‐S‐transferase, allowing the scavenging of free radicals in cells caused by oxidative damage.^30^ As shown in Figure 6A, in the cytoplasm, PIPO significantly decreased the LPS‐stimulated protein expression of cytosolic Nrf2. However, PIPO activated the LPS‐stimulated protein translocation from the cytoplasm into the nucleus and increased the protein expression of Nrf2 in the nucleus (Figure 6A). Moreover, PIPO could upregulate the antioxidative protein expressions of HO‐1, CAT, and SOD 1 & 2 (Figure 6B). Furthermore, using the immune‐fluorescence analysis showed that PIPO could decrease the protein expression of Nrf2 in the cytoplasm but increase translocation and accumulation of Nrf2 in the nucleus (Figure 6C). The collective data indicate that PIPO promoted the nuclear translocation of Nrf2 from the cytoplasm into the nucleus and upregulated the antioxidative protein expressions.

### 3.7 PIPO suppressed the TLR4‐MyD88 pathway in LPS‐stimulated RAW264.7 cells

**FIGURE 7:**
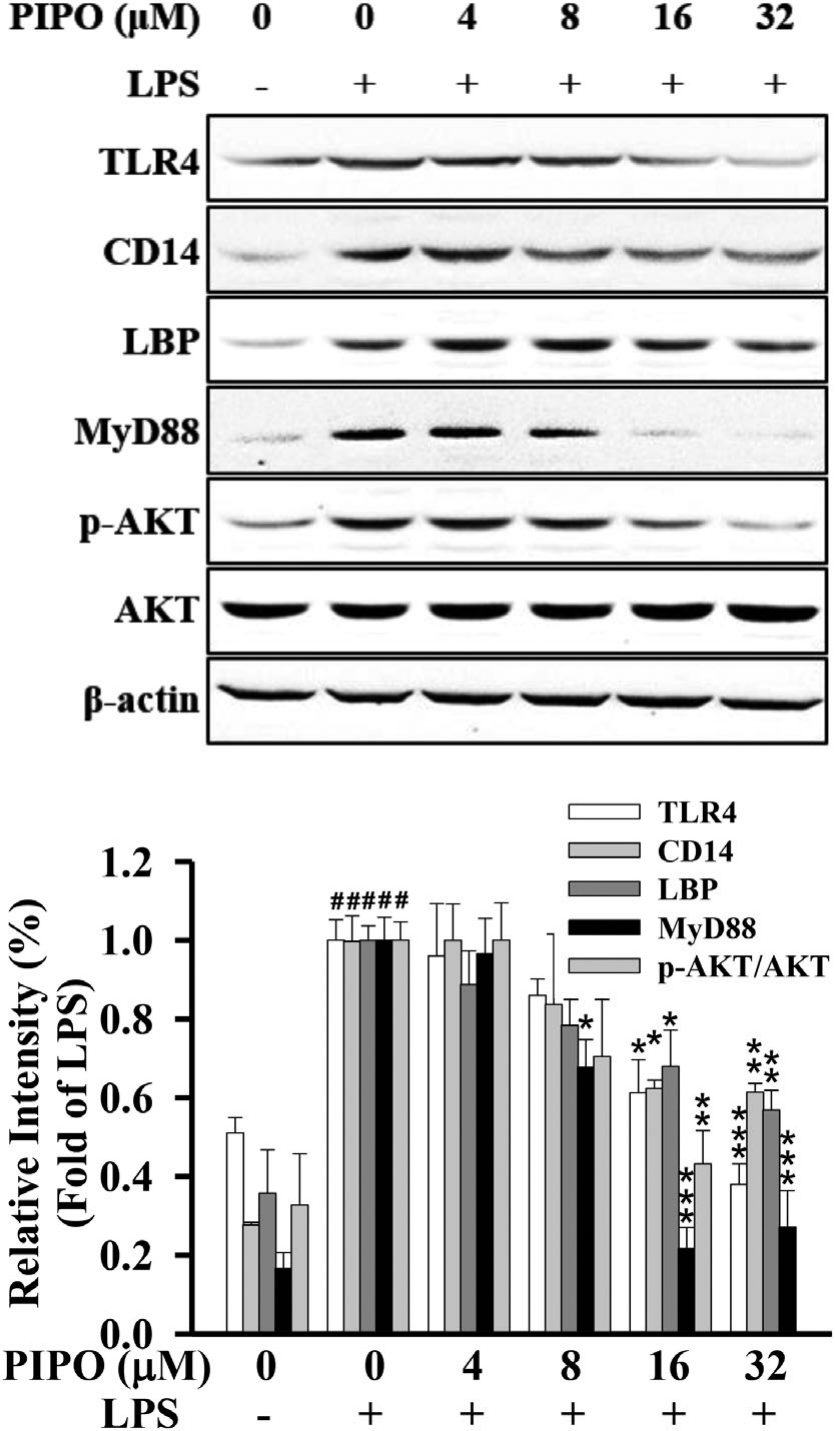
Pipoxolan (PIPO) suppressed the TLR4‐MyD88 pathway in lipopolysaccharide (LPS)‐stimulated RAW264.7 cells. (A) RAW264.7 cells were pretreated with PIPO (0, 4, 8, 16, and 32 μM) for 1 hour and then stimulated with LPS (100 ng/mL) for 30 minutes. The phosphorylation of AKT was measured by western blotting (n = 3). The protein expression of TLR4, BLP, CD14, and MyD88 were measured by western blotting. RAW264.7 cells were pretreated with PIPO (0, 4, 8, 16, and 32 μM) for 1 hour and then stimulated with LPS (100 ng/mL) for 24 hours. Data are presented as mean ± SE. #*P* < .05 compared with the negative LPS‐stimulated group, *P < .05, **P < .01, and ***P < .001 compared with the LPS group

LPS bind to LPS‐binding protein (LBP) and then transfers and binds to the Cluster of differentiation 14 (CD14) and load the LPS to the TLR4‐bound complexing coreceptor MD‐2.^2^ As shown in Figure 7, PIPO significantly inhibited the protein expression of LBP, CD14, MD‐2, and TLR4 on the cell membrane in LPS‐stimulated RAW264.7 cells (Figure 7). The present finding supports that PIPO applies anti‐inflammatory activity through the modulation of the TLR4/p‐AKT signaling pathways.

## 4 DISCUSSION

Inflammation is an essential immune response defense process of the body against the stimulus, infection, or injury. However, chronic inflammation also causes immune deficiency and promotes the development of many diseases or cancer.^31^ LPS/TLR4 stimulates the inflammatory transcription factor signaling pathway (including NF‐κB and AP‐1 transcription factors) and causes the release of pro‐inflammatory factors (iNOS, COX‐2).^32^ Therefore, overstimulation of the NF‐κB transduction‐signaling pathway thus produce a pathogen‐specific essential immune response and release pro‐inflammatory cytokines (TNF‐α, IL‐6, and IL‐1β).^33^ In the present study, we found that PIPO effectively inhibited LPS‐stimulated production of cytokine nitrite and PGE_2_, which confirmed the protein and mRNA expression of the upstream pro‐inflammatory mediator iNOS and COX‐2. Besides, PIPO also reduced the production of cytokine TNF‐α and IL‐6 in LPS‐stimulated RAW264.7 cells.

In general, NF‐κB always stabilized in the cytoplasm by binding to the IκB junction.^26^ Two related kinases, IKKα and IKKβ, phosphorylate the IκB proteins, prime to their degradation and the subsequent stimulation of gene expression of NF‐κB.^34^ The translocation of the NF‐κB p50‐p65 dimer to the nucleus promotes the release of inflammatory mediators such as TNF‐α, IL‐6, IL‐1β, NO, and iNOS.^35^ Our results suggested that PIPO significantly inhibited LPS‐induced phosphorylation of IκB and decreased the protein expression of transcription factor NF‐κB (p65 and p50) and reversed the nuclear translocation of NF‐κB in LPS‐stimulated RAW264.7 cells. The results were confirmed by the immunofluorescence analysis in the cytoplasm and nucleus. As a result, PIPO inhibited the NF‐κB pathway participated in the anti‐inflammatory process.

However, p38 MAPK induced expressions of TNF‐α, IL‐1β, IL‐6, and IL‐8.^36^ Therefore, our results showed that PIPO could inhibit MAPK signaling by decreasing the phosphorylation of JNK, ERK1/2, and p38 and reducing the nuclear translocations of c‐Jun and c‐Fos from the cytoplasm into the nucleus in LPS‐stimulated RAW264.7 cells. It has been demonstrated that TNF‐α as the critical trigger along with increased activation of JAK1/STAT1 plays a crucial role in tumor cell proliferation and invasiveness.^37^

In particular, IL‐6 is the primary mediator of inflammation, which is the key to trigger the JAK2/STAT3 pathway, and plays a crucial role in tumor cell proliferation and invasiveness.^38^ This study shows that PIPO inhibited the cytokine TNF‐α and IL‐6 production, and PIPO is effective in reducing the phosphorylation and the nuclear translocations of JAK1, 2/STAT1, 3. This study assessed whether the anti‐inflammatory function of PIPO is associated with the inhibitions of the JAK/STAT pathway and participated in the anti‐inflammatory process.

**FIGURE 8:**
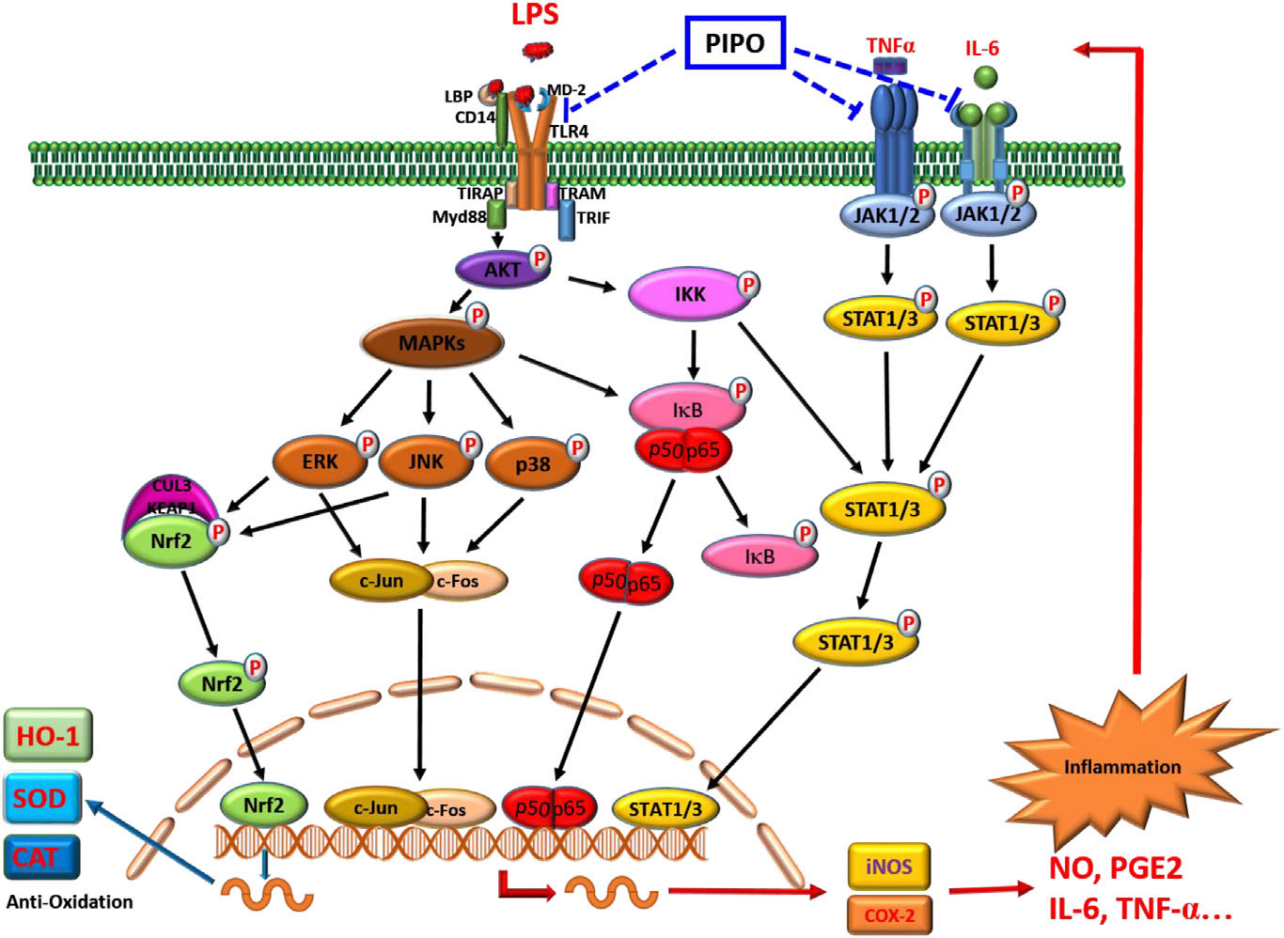
Proposed mechanism depicting the effect of pipoxolan (PIPO) suppressing the TLR4/AKT pathway, which inhibited the anti‐inflammatory effects via the mitogen‐activated protein kinase (MAPK)/AP‐1, inhibitor kappa B (IκB)/NF‐κB, and Janus kinase (JAK)/signal transducer and activator of transcription (STAT) transcription factor pathways, and activating the antioxidant effects via Nrf2/HO‐1 signaling pathways on lipopolysaccharide (LPS)‐stimulated macrophages [Color figure can be viewed at wileyonlinelibrary.com]

Interestingly, the oxidative stress pathway is the essential mechanism of the inflammatory response. Nrf2 plays a critical role in oxidative stress.^39^ TLR4/MyD88 signaling can activate Nrf2 but independent from the reactive oxygen species.^40^ Under the stimulation of oxidative stress, Nrf2 translocated from the cytoplasm into the nucleus, stimulating the transcription of antioxidant response element‐dependent genes, including HO‐1, CAT, and SOD.^12^ The data showed that PIPO attenuates the inflammatory effects via influencing LPS/TLR4 pathway, which significantly suppressed the anti‐inflammatory transcription factors NF‐κB, AP‐1, and STAT and activates the antioxidative transcription factor Nrf2 in LPS‐stimulated RAW 264.7 cells (Figure 8).

In conclusion, our data demonstrate that PIPO attenuates the inflammatory effects via influencing the LPS/TLR4 pathway; suppresses the anti‐inflammatory transcription factor NF‐κB, AP‐1, and STAT; and activates the antioxidative transcription factor Nrf2 in LPS‐stimulated RAW 264.7 cells.

## CONFLICT OF INTEREST

The authors declare no potential conflict of interest.

## AUTHOR CONTRIBUTIONS

W.‐T.H. and Y.‐P.L. designed the research. Y.‐H.L., Y.‐J.L., and T.‐H.C. conducted anti‐inflammatory experiments. Y.‐H.L. wrote the manuscript. Y.‐H.C., J.‐G.C., and W.‐T.H. revised the manuscript. All authors reviewed the manuscript.
